# Dr. Thaddeus Asbury Reamy

**Published:** 1909-04

**Authors:** 


Dr. Thaddeus Asbury Reamy, of Cincinnati, died in that city March 11, 1909, aged 79 years. He graduated from Starling Medical College, Columbus, Ohio, in 1854, and received the degree of arts from Ohio Wesleyan University, 1868; and LL.D., from Cornell in 1889. He began teaching in the Cincinnati College of Medicine and Surgery in 1858. He was a pioneer in the field of gynecology in the middle west, became president of the American Gynecological Society in 1885, and obtained extensive membership in medical organisations besides being a large contributor to the literature of his specialty.

